# Genetic Heterogeneity of Single Circulating Tumour Cells in Colorectal Carcinoma

**DOI:** 10.3390/ijms21207766

**Published:** 2020-10-20

**Authors:** Faysal Bin Hamid, Vinod Gopalan, Marco Matos, Cu-Tai Lu, Alfred King-yin Lam

**Affiliations:** 1 Cancer Molecular Pathology, School of Medicine, Griffith University, Gold Coast, QLD 4222, Australia; faysal-bin.hamid@griffithuni.edu.au; 2 Oncology, Gold Coast University Hospital, Gold Coast, QLD 4215, Australia; marco.matos@health.qld.gov.au; 3 Colorectal Surgery, Gold Coast University Hospital, Gold Coast, QLD 4215, Australia; cutlu01@yahoo.com

**Keywords:** CTC, single-cell analysis, gene expression, heterogeneity, colorectal carcinoma

## Abstract

The aim of the present study was to isolate and investigate the genetic heterogeneities in single circulating tumour cells (CTCs) from patients with colorectal carcinoma (CRC). Twenty-eight single CTCs were collected from eight patients with CRC using a negative immunomagnetic enrichment method. After validation with glyceraldehyde 3-phosphate dehydrogenase (GAPDH) gene expression in 3 colon cancer cell lines, a panel of 19 genes were used to analyse the single CTCs (*n* = 28), primary colorectal carcinoma tissues (*n* = 8) and colon carcinoma cells (*n* = 6) using real-time qPCR. Genetic heterogeneities were assessed by comparing gene expression profiles of single CTCs from the different patients and in the same patient, respectively. Genetic profiling of the single CTCs showed extensive heterogeneities of the selected genes among the CTCs. Hierarchical clustering analyses exhibited two clusters of CTCs with differentially expressed genes, which highlighted different modifications from the primary carcinomas. Further, the genetic heterogeneities were observed between different patients or in the same patient. Finally, *AKT1* expression was significantly (*p* = 0.0129) higher in single CTCs from CRC of advanced pathological stages (III or IV) CRC than in CTCs from CRC of early stages (I or II). Our findings suggest that single-cell genetic analysis can monitor the genetic heterogeneities and guide the personalised therapeutic targets in clinical sectors.

## 1. Introduction

Circulating tumour cells (CTCs) have been considered as a minimally or non-invasive diagnostic and prognostic biomarker in patients with colorectal carcinoma (CRC) [1,2]. Previous studies have shown that CellSearch could isolate only epithelial CTCs [3,4,5]. In addition, other CTCs, including mesenchymal CTCs with higher metastatic potential, which comprise only 0.01% of the total CTC population, could evade immunomagnetic assays [5]. Thus, the clinical relevance of heterogeneous CTCs is not well understood.

Many groups have confirmed the presence of heterogeneous CTC phenotypes (such as epithelial, epithelium–mesenchymal transition (EMT) and mesenchymal/stem) in patients with different cancers, including CRC [6,7]. Interestingly, immunoaffinity-dependant negative selection techniques demonstrated higher efficacy in isolating heterogeneous CTCs from the blood of patients with cancer [8,9]. Molecular characterisation of the heterogeneous CTCs showed that they were more concordant with metastatic tumours than primary tumours in patients with colorectal cancers, suggesting the acquired characteristics of the CTCs in the blood microenvironment [10].

The genetic characterisation of enriched CTCs could exhibit the global molecular portrait of the metastatic microenvironment [11,12,13,14]. However, it is unlikely to reveal the role of individual cancer cells due to the presence of other contaminated blood cells [1,10]. Besides, single-cell analysis requires a highly sensitive workflow to isolate the single CTCs from millions of lymphocytes and subsequent downstream experiments. Previous studies have shown that genetic profiles of single CTCs could display a comprehensive landscape of the molecular aberrations in the different signalling pathways and pinpoint new therapeutic targets [11,12,14]. Moreover, single-cell analysis of CTCs can exhibit discrepancies in the gene expression in different patients and within individual patient. This can further indicate systemic therapy ineffectiveness and relapse of the disease [15].

In the present study, we isolated 28 CTCs from 8 patients with colorectal carcinoma using a negative selection method. In addition, we compared genetic profiles of single CTCs with the primary tumour tissues and evaluated the genetic heterogeneities in different patients and within the same patient. Finally, we correlated the gene expression patterns with the pathological stages of the patients with colorectal carcinoma.

## 2. Results

### 2.1. Validation of the Real-Time qPCR Experiment at the Single-Cell Level

**Figure 1:**
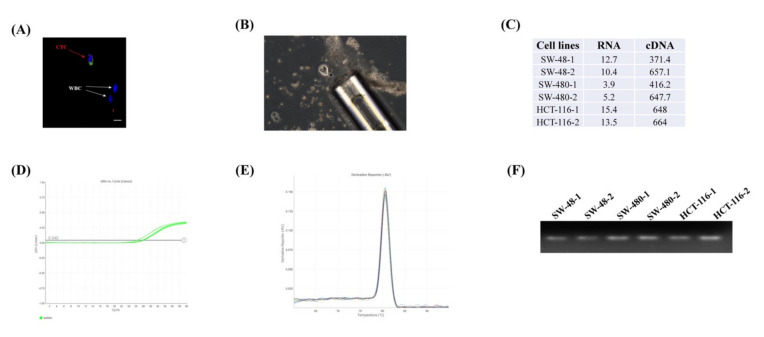
Assessment of the gene expression profiling from the single cell. (**A**) Identification of the circulating tumour cell (CTC) from the other blood cells e.g. white blood cells (WBCs) (**B**) Isolation of single CTC. (**C**) The concentration and cDNA concentration (ng/μL) of the six cells from three colon cancer cell lines (SW-48, SW-480 and HCT-116). The RNA integrity was carried out by assessing the gene expression of glyceraldehyde 3-phosphate dehydrogenase (GAPDH) showing (**D**) Ct values of < 30 and (**E**) a single peak in the melting curve. (F) PCR product was run in 2% agarose gel to confirm the qPCR results.

To validate the quantitative gene expression from the single cell, we initially tested the concentration and integrity of the extracted RNAs from the single cells (*n* = 2) of three colon cancer cell lines. The concentrations of the RNA samples ranged from 3 to 15 ng/μL in the Nanodrop 2.0 system (Figure 1C). We also evaluated RNA integrity using a qPCR experiment of the highly expressed endogenous gene glyceraldehyde 3-phosphate dehydrogenase (GAPDH) (Figure 1D). The Ct value was < 30, and a single peak in the melt curve analysis for each sample suggested that the RNA was intact, of good quality and suitable for PCR performance (Figure 1E). The PCR product was also confirmed by running in 2% agarose gel (Figure 1F).

### 2.2. Multi-marker mRNA Profiling of the Single CTCs, Matched Tumours and Colon Cancer Cell Lines

**Figure 2:**
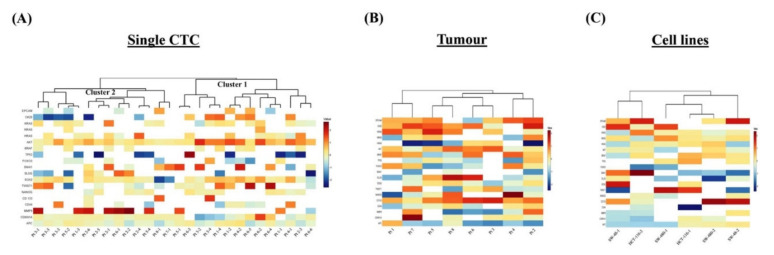
Single-cell genetic analyses of the CTCs, primary carcinomas and colon carcinoma cell lines. Heatmap of the heterogeneous expression of 19 genes from (**A**) single CTCs (*n* = 28), (**B**) primary carcinoma (*n* = 8) and (**C**) single cells (*n* = 2) from each of the three colon carcinoma cell lines.

To determine the diverse gene expression profiles of the 28 single CTCs and the corresponding 8 primary tumours, we evaluated relative expression levels of 19 genes of different functions. The relative expression (RQ) status demonstrated an intricate pattern of the gene expressions among the single CTCs (Figure 2A). Unsupervised hierarchical clustering analysis showed that the gene expressions of the single CTCs were grouped into two clusters: clusters 1 and 2. Expressions of *AKT1, CDKN1A* and *APC* were found in both clusters of CTCs. Interestingly, the expressions of the epithelial and tumour suppressor genes were lower, and *HRAS, SOX2* and *MMP9* were higher in cluster 1. In cluster 2, the epithelial marker *CK20* and the stemness gene *SNAI1* were overexpressed, but *CD44, CDKN1A* and *APC* were under-expressed. Further, the gene expression profiling of the tumour exhibited a higher level of *EPCAM, CK20, KRAS, AKT1, BRAF* and *CD133* expression, while lower levels of expression were observed in *NANOG, MMP9* and *APC* genes (Figure 2B). Besides, the gene expression of the cell lines showed differential patterns such as overexpression of *EPCAM, CK20* and *CD133* genes and lower levels of expression of *SNAI1* and *APC* genes (Figure 2C).

**Figure 3:**
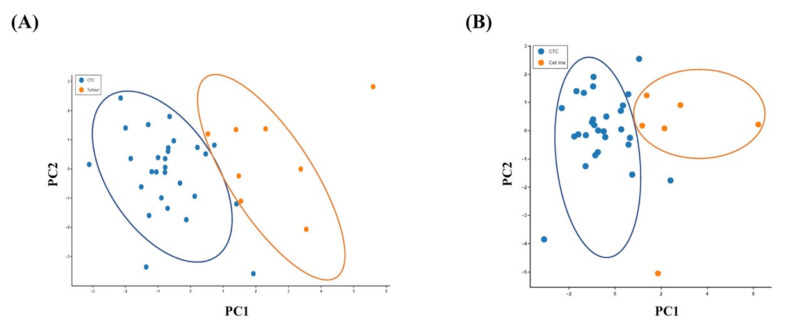
Relationship of the gene expressions of the CTCs with the primary carcinoma and the colon cancer cell lines. Principal component analysis (PCA) plot of the expression of 19 genes in (**A**) single CTCs and the primary carcinomas and (**B**) single CTCs and the colon cancer cells in cell lines.

Principal component analysis (PCA) was performed to visualise the variability of the single CTCs from the original tumours and the colon cancer cell lines. A PCA plot derived from normalised gene expression data showed a clear separation of the CTC population from the primary tumour (Figure 3A) or the cancer cell in cell lines (Figure 3B). It revealed distinct genetic patterns in the single CTCs, indicating that CTCs use distinct biological alterations to survive in the blood circulation compared with cancer cells in the matched primary tumour tissues and viable cancer cells.

### 2.3. Genetic Heterogeneities of the Single CTCs

**Figure 4:**
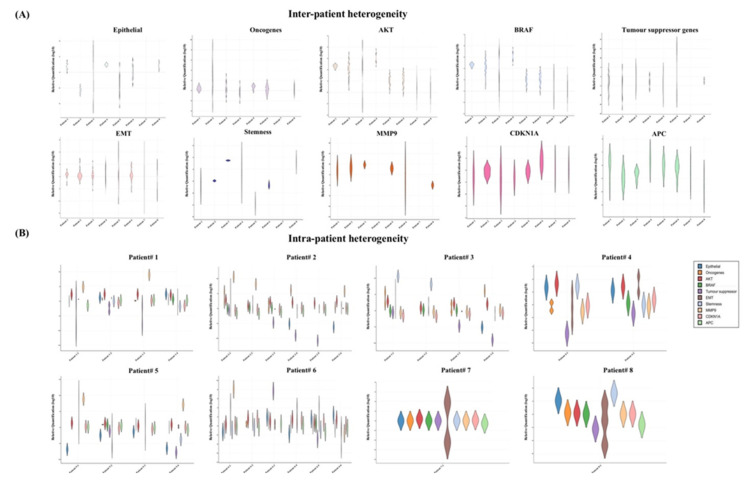
Gene expression heterogeneities of the single CTCs in different patients and in the same patient. Violin plot of the gene expression profiles in the single CTCs showed heterogeneities (**A**) in different patients and (**B**) in the same patient.

Next, we analysed the gene expression profiles of the single CTCs, which were exclusively varied from different patients and even within the same patient (Figure 4). For instance, the gene expression profiles of the eight patients showed that the epithelial markers were overexpressed in >50% in the CTCs in three out of the eight patients (patients 1, 4 and 8) (Figure 4A). On the other hand, the markers were expressed at lower levels in only patients 2 and 5. Similarly, stem-cell-like properties were observed at higher levels in patients 3, 4 and 8 and downregulated in patients 1, 5 and 6. Gene expression patterns of the oncogenes and tumour suppressor genes were apparently antagonistic in the CTCs. Tumour suppressor genes were expressed at lower levels in patients 1, 2, 4 and 5, while upregulation of the oncogenes was found in patients 1, 2, 3 and 5. Interestingly, EMT genes and CDKNA1 were differentially expressed. Overall induction of *AKT1* and *MMP9* was observed in the total patient cohort.

We also found different gene expression patterns in the individual patients indicating the presence of the heterogeneous CTC subclones (Figure 4B). For example, in patient 1, we found two types of CTCs: epithelial-marker-positive and epithelial-marker-negative CTCs. Among the epithelial CTCs, the tumour suppressor genes were only expressed in the *EPCAM+* CTC, not in *CK20+* CTCs. In addition, the presence of the extracellular matrix (ECM)-degrading gene *MMP9* was merely observed in the epithelial-marker-negative CTCs in two patients (patients 1 and 5). In patient 2, the epithelial marker was either downregulated or absent. Like n patient 1, two of the epithelial-negative CTCs in patient 2 expressed a higher level of *MMP9*. Further, the expressions of *MMP9* and the tumour suppressor genes were likely opposite in patient 2. In patients 3 and 4, CTCs expressed only *CK20*+, where at least two CTCs were positive for *CD44*. However, CTCs of patient 5 expressed only *CK20* at a lower level with constant positivity of *SLUG, SOX2* and *TWIST1*. Interestingly, one CTC with *EPCAM-/CK20-/CD44+* in patient 5 indicated the apparent existence of the mesenchymal CTCs. In patients 6 and 8, the co-expression of epithelial and EMT genes in the CTCs was observed, but the distribution of the stemness markers was different. We found the presence of *CD44* and *CD133* in patients 6 and 8, respectively, while none of the epithelial or stemness markers was found in patient 7.

### 2.4. Correlation of the Gene Expression of Single CTCs and the Tumour Stages

**Figure 5:**
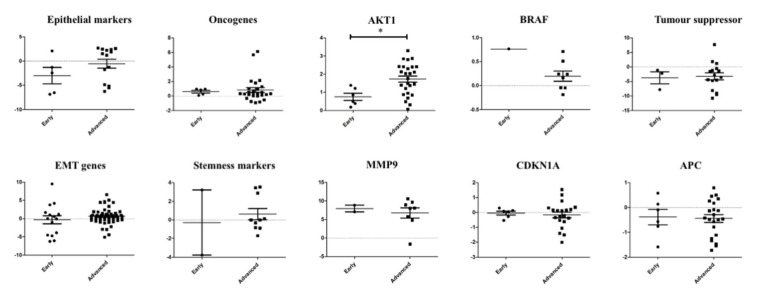
Correlation of the gene expression between single CTCs and pathological stages of colorectal carcinoma. Comparison of the gene expression of the single CTCs in colorectal carcinoma of early and advanced stages showed that only expression of *AKT1* was associated with pathological stages of the cancer.

To investigate the role of the genes triggering metastasis, we analysed the association of the gene expression of the single CTCs with the pathological staging (early stage—I or II; advanced stage—III or IV) of colorectal carcinoma (Figure 5). The genetic profiling showed the involvement and different alterations of the several signalling pathways in the CTCs—for example, the EMT pathway, the P53 signalling pathway and the Wingless-related integration site (WNT) signalling pathway. In the CTCs from CRC of advanced stages, epithelial markers, cell survival (*AKT1*), tumour suppressor and stemness genes were overexpressed. In contrast, cell proliferation gene (*BRAF*), ECM-degrading gene (*MMP9*), cell cycle regulating and WNT signalling pathway regulator (*APC*) were expressed higher in the cancers of early pathological stages. Among them, only *AKT1* expression was found to be significantly (*p* = 0.0129) correlated with progression of cancers.

## 3. Discussion

Over the past decades, CTCs have been considered as a potential candidate for the “liquid biopsy” since they act as “snapshot” of the primary tumour [16,17]. Recent studies showed that heterogeneous CTCs were often detected from the blood and were phenotypically and genetically different from the primary tumour [18,19,20]. The heterogeneity of the CTCs can be used in real-time monitoring of the disease, which could add value to the gold standard “tumour biopsy”. Interrogating each CTC at once, therefore, could be useful to study tumour heterogeneity and identify new therapeutic targets leading to personalised treatment [20]. In the present study, we examined the gene expression of the CTCs at a single-cell resolution with comparison to the matched primary tumour tissues.

To date, several studies have been published on the molecular characterisation of single CTCs from different cancers, such as prostate, pancreatic and colorectal carcinomas [9,20,21]. In the current study, the single-cell quantitative PCR performance was validated by gene expression of GAPDH from the colon cancer cell lines. Unsupervised clustering analyses and PCA plots demonstrated that the expression patterns of the genes were different among the single CTCs and their corresponding primary tumours. The results imply that CTCs, unlike the matched primary tumours, have distinct patterns of heterogeneity at the genetic level.

The gene expression of single CTCs displays various insights into the modifications that occur in the lifecycle of CTCs [22,23]. Involvement of the immune evasion and survival pathways was shown in the CTCs from colorectal carcinoma [14]. We found lower expressions of the epithelial genes in the CTCs compared with the primary tumour samples. According to the canonical metastasis model, CTCs are required to alter morphological characteristics such as the disappearance of the cell adhesion molecules (EPCAM), rearrangement of the cytoskeletons and degradation of the ECM [24]. Previous studies showed that CK20 expression was associated with the prognosis of patients with colorectal carcinoma [25,26]. Simultaneously, differential expressions of the EMT and stemness markers were also observed among the CTCs. However, co-expression of the EMT and stemness genes indicated that some EMT-expressing CTCs had stem-cell-like or tumour-initiating properties.

Interestingly, we observed a lower level of tumour suppressor genes and a higher level of the oncogenes in CTCs, especially *HRAS*, which is involved in cell motility [27]. Moreover, the tumour suppressor gene *TP53* can directly induce initiation and progression by activation of a series of transcription factors involved in metastasis, such as *SNAI1*, *SLUG*, *NANOG* and so forth, resulting in EMT formation [28]. In addition, loss of *TP53* is associated with the induction of the proteolytic enzyme MMPs that disrupt cell–ECM interaction and enhance stemness marker *CD44* [29,30]. Dhar et al. demonstrated that *MMPs* could be expressed approximately 200 times higher in the CTCs [31]. Similarly, we observed that MMP9 expression was higher in the CTCs, which indicates their increased ability to degrade the ECM or basal matrix during intravasation or extravasation.

Furthermore, *AKT* can induce EMT, which leads to cell survival, motility and invasion in colorectal cancer. Further, our results showed that *AKT1* expression was significantly (*p* = 0.0129) increased in CRC of advanced stages. Overexpression of *AKT1* augments *CD133* expression, leading to higher chemotherapeutic resistance [32]. On the other hand, higher *AKT1* expression can downregulate *CD44* and apoptosis inhibitory genes in the P53 signalling pathway, which provides higher cell survival to the CTCs in colorectal carcinoma.

AKT1 has also been associated with the APC–CDKN1A network [33]. Our results showed the negative expression of the cell cycle regulator CDKN1A and WNT signalling pathway regulator APC in a subset of the CTCs, suggesting the involvement of the canonical WNT pathway in colorectal carcinoma. Downregulation of APC resulted in the accumulation of beta-catenin in the nucleus and increased transcription of the downstream genes associated with the development of colorectal carcinoma [34]. The involvement of the noncanonical WNT signalling pathway was reported in pancreatic CTCs, and RNA sequencing data suggest WNT2 as a candidate of therapeutic target [35].

Colorectal carcinoma often relapses and metastasises to the secondary organs, mainly the liver and lungs [36]. The failure of systemic or neoadjuvant therapies in treatments of CRC could be related to the heterogeneities of CTCs [37]. Therefore, understanding the heterogeneity among the CTCs could suggest more precise therapeutic targets for different subclones of cancer cells. Likewise, we found that the gene expression profiles of the CTCs were heterogeneous in different patients as well as within the same patient. Several studies have reported genetic heterogeneities of levels of CTCs in different patients or in the same patient in different carcinomas, such as pancreatic, breast, ovarian and prostate carcinomas [12,37,38,39]. Our results demonstrate that most of the patients had both differentially expressed epithelial and EMT genes despite only patients 3 and 8 having a higher level of the stemness genes. Moreover, one CTC from patient 3 and one from patient 4 were positive for CD44. CD44+ cells are known as tumour-initiating cells. Hence, analysis of single CTCs could direct clinicians to the precise treatment for individuals, leading to personalised treatment.

## 4. Materials and Methods

### 4.1. Cell Culture

The human colon cancer cell lines (SW-48, SW-480 and HCT-116) were purchased from the American Type Culture Collection (ATCC, Manassas, VA, USA). The cell lines were cultured in Roswell Park Memorial Institute (RPMI) 1640 medium (Gibco, Carlsbad, CA, USA) containing 10% foetal bovine serum (FBS) (Gibco) and 1% penicillin/streptomycin (Gibco) in a humidified 37 °C, 5% CO_2_ incubator.

### 4.2. Patient Enrolment and Sample Processing

Eight patients with CRC were included in the study who had CTCs in their blood and were confirmed by histopathological examination. Three of the eight patients were diagnosed with early stage (2 stage I and 1 stage II) CRC, and five patients were diagnosed with advanced pathological stage (2 stage III and 3 stage IV) CRC. The ethics of the study was approved by the Griffith University Human Research Ethics Committee (GU Ref No: MSC/17/10/HREC). All patients signed a written informed consent form before participation in the study. The clinical parameters (i.e., age, gender, sex, etc.) and pathological data (tumour size, stage, etc.) of the patients were recorded after pathological examination of the resection of the colorectal carcinoma and were unknown during the experiments. On the day of resection, tumour specimens and blood samples were freshly collected from the patient. The blood and tumour samples were processed within 1 h of collection. The fresh tumour specimens were snap-frozen by liquid nitrogen, preserved in RNA*later* to avoid RNA degradation and stored in a −80 °C freezer until further use.

### 4.3. CTC Enrichment and Identification

In this study, we used EasySep^TM^ Direct Human cell isolation (STEMCELL Technologies., Vancouver, BC, Canada) to enrich CTCs from the whole blood of the patients. In brief, 5 mL of freshly collected blood was incubated with a cocktail of antibody-labelled magnetic beads for 10 min, followed by mixing the samples with the “enrichment cocktail” (buffer provided by company). The ferrofluidic beads can target CD2-, CD14-, CD16-, CD19-, CD45-, CD61-, CD66b- and glycophorin-A-harbouring blood cells and eliminate them when the blood containing tube is placed on the EasySep^TM^ magnet platform (STEMCELL Technologies). By rapid inversion, the suspension was transferred and centrifuged to precipitate the remaining cells. Then, the enriched CTCs were placed in a 12-well culture plate and incubated overnight.

CTCs were identified by positive staining of Hoechst 33342 and a cocktail of four antibodies as following: mouse EPCAM (Thermo Fisher Scientific, Waltham, MA, USA), CK-18 (Abcam, Cambridge, UK), E-cadherin (Santa Cruz Biotechnology, Santa Cruz, CA, USA), and goat SNAI1 (Santa Cruz Biotechnology) (Figure 1A). In brief, the enriched cells were permeabilised with 0.2% Triton X-100 for 10 min, followed by fixing with 100% methanol at −20 °C. After washing with phosphate-buffered saline (PBS) three times, the cells were incubated with the four primary antibodies and 1% bovine serum albumin (BSA) for 2 h and subsequently with corresponding secondary anti-mouse FITC (green signal) and anti-goat Texas Red (red signal) (Sigma Aldrich, St. Louis, MO, USA) antibodies for 1 h. Then, the cells were incubated with Hoechst 33342 (blue signal) (Thermo Fisher Scientific) for 5 min. Finally, the cells with three positive signals were counted as CTCs under a fluorescent microscope (Olympus, Tokyo, Japan).

### 4.4. Single CTC Isolation, RNA Extraction and Complementary DNA (cDNA) Conversion

From 8 patients (5 metastatic and 3 nonmetastatic) with CRC, 28 single CTCs were isolated. We used six single colon cells from three different cell lines as a control. Single CTCs were collected with a stripper micropipetter (Origio, Målov, Denmark) with 50 µm tips under a microscope and were transferred into a 96-well plate (Figure 1B). Total RNA from the single CTCs was extracted using the Single Cell Purification Kit (Ref. 51800, Norgen Biotek Corp, Thorold, ON, Canada) according to the manufacturer’s instructions [40]. In brief, a lysis buffer RL was immediately added to the freshly isolated single CTCs to prepare the cell lysate. The lysate was immediately transferred to the spin column for binding the RNA followed by adding 70% ethanol. The bound RNA was washed, and the final RNA was eluted in 8 µl of elusion buffer. The concentration and the quality of the RNAs were measured by the Nanodrop 2.0 system (Thermo Fisher Scientific).

Reverse transcription (RT) was carried out by the Sensiscript RT kit (Qiagen, Hilden, Germany) from the total RNA of the single CTCs according to the manufacturer’s instructions. A total of 20 µL of RT mixture composed of 1× of 10× Buffer RT, 0.5 mM each dNTPs, 10 µM oligo-dT primer, 1 µL of Sensiscript Reverse Transcriptase and 8 µL of total RNAs was incubated for 1 h at 37 °C. The cDNAs were stored at −20 °C for further use.

### 4.5. RNA Extraction from Frozen Tumour Tissues and Reverse Transcription

Total RNA was extracted from the tumour specimens using the RNeasy mini kit (Qiagen) as described previously [41]. Twenty milligrams of frozen tumour tissues were sectioned into 10 µm slices to extract the total RNA. Total RNAs were eluted in 50 µL of RNase-free water and stored at −80 °C. Five microlitres of total RNAs were used to make the cDNAs using the QuantiTect reverse transcription kit (Qiagen). The RNA samples were incubated with gDNA wipeout buffer at 42 °C for 2 min to avoid DNA contamination. Twenty microliters of the reactions were performed by incubating at 42 °C for 15 min and stopped at 95 °C for 3 min.

### 4.6. Quantitative Real-Time Polymerase Chain Reaction (qRT PCR)

Quantitative gene expression of the 19 genes was performed using the QuantiTect SYBR Green PCR kit (Qiagen). The selected genes were acquired from GeneCards database (https://www.genecards.org) and categorised according to the functions. Primers were designed using Primer3 and purchased from Sigma Aldrich (St. Louis, Missouri, USA). The sequences of the genes are described in Table 1.

GAPDH was used as an internal control for normalisation. In brief, a total of 10 µL of the assays contained a final concentration of 5 µL of SYBR Green PCR master mix, 0.5 µM of each primer and 60 ng/µL of the diluted cDNA. In a QuantStudio 6 Flex Real-Time PCR system (Applied Biosystems, Foster City, CA, USA), the reaction mixtures were amplified in 40 cycles: 95 °C for 15 min, 94 °C for 15 s, 54–60 °C for 30 s and 72 °C for 30 s. The relative gene expression levels were calculated using the delta–delta Ct method as described previously [36,42]. Leukocytes from healthy persons were used as a control.

Gene expression profiles were visualised as heatmaps and the variabilities of the gene expressions of the single CTCs against the primary tumours and colon cell lines were displayed as principal component analysis (PCA) plots. The gene expressions of the single CTCs in each patient and total patients’ group were compared to evaluate the intra- and inter-patient heterogeneity and were exhibited with violin plots.

### 4.7. Statistical Analyses

The statistical analyses were executed by Student’s *t* test using GraphPad Prism Software 5.03 (GraphPad Software Inc., San Diego, CA, USA). Results from qPCR experiments were presented as mean ± SEM. Statistical significance was analysed using the Mann–Whitney test. The data were considered significant at *p* < 0.05. Log10 values of the relative gene expression levels were plotted to generate the heatmaps using complete linkage clustering and the Euclidean distance method. Similarly, PCA plots were generated with log10 transformation of the data with a 95% confidence interval. Heatmap, PCA and violin plots were generated with BioVinci software version 1.1.0 (BioTuring Inc., San Diego, CA, USA).

## Figures and Tables

**Table 1:** Primer list.

	Genes	Accession no.	Forward (5ʹ-3ʹ)	Reverse (5ʹ-3ʹ)	Amplicon Size (*bp)
Epithelial	EPCAM	NM_002354.3	CGCAGCTCAGGAAGAATGTG	TGAAGTACACTGGCATTGACG	88
	CK20	NM_019010.3	CAGTCCCATCTCAGCATGAAAG	CCTCCAGAGAGCTCAACAGC	109
Oncogenes	KRAS	NM_033360.3	AGGCCTGCTGAAAATGACTGAATAT	GCTGTATCGTCAAGGCACTCTT	80
	NRAS	NM_002524.4	TTGAGGTTCTTGCTGGTGTG	TTAGCTGGATTGTCAGTGCG	92
	HRAS	NM_005343.4	TACCGGAAGCAGGTGGTCAT	GATGGCAAACACACACAGGA	135
Proliferation	BRAF	NM_004333.6	TCTTCATGAAGACCTCACAGT	CCAGACAACTGTTCAAACTGA	96
Cell survival	AKT1	NM_005163.2	AAGTACTCTTTCCAGACCC	TTCTCCAGCTTGAGGTC	197
Tumour suppressor	TP53	NM_000546.5	ACCTATGGAAACTACTTCCTG	ACCATTGTTCAATATCGTCC	99
	FOXO3	NM_001455.4	GAATGTTGTTGGTTTGAACG	ATTTGGCAAAGGGTTTTCTC	156
*EMT	SNAI1	NM_005985.4	AACAATGTCTGAAAAGGGAC	ATAGTTCTGGGAGACACATC	95
	SLUG	NM_003068.5	CATGCCTGTCATACCACAAC	GGTGTCAGATGGAGGAGGG	169
	SOX2	NM_003106.4	GATCCTGGACTTCTTTTTGG	TCTATACAAGGTCCATTCCC	87
	TWIST1	NM_057179.3	ATCATTTGTAACAACCCAGG	CAAATGATAGAGTCAGCACC	160
	NANOG	NM_024865.4	CTATCCATCCTTGCAAATGTC	GTTCTGGTCTTCTGTTTCTTG	198
Stemness	CD133	NM_006017.3	CACTACCAAGGACAAGGCGTTC	CAACGCCTCTTTGGTCTCCTTG	151
	CD44	NM_000610.4	CCAGAAGGAACAGTGGTTTGGC	ACTGTCCTCTGGGCTTGGTGTT	151
*ECM-degrading	MMP9	NM_004994.3	AAGGATGGGAAGTACTGG	GCCCAGAGAAGAAGAAAAG	151
Cell cycle regulator	CDKN1A	NM_000389.5	CAGCATGACAGATTTCTACC	CAGGGTATGTACATGAGGAG	200
*WNT pathway regulator	APC	NM_001127511.3	AGAGGTCATCTCAGAACAAG	CATGTTGATTTCTCCCACTC	86
Housekeeping	GAPDH	NM_002046.7	TGCACCACCAACTGCTTAGC	GGCATGGACTGTGGTCATGAG	87

* bp = Base pair, EMT = Epithelial–mesenchymal transition, ECM = Extracellular matrix.

## Abbreviations

CTCs	Circulating tumour cells
CRC	Colorectal carcinoma
qPCR	Quantitative polymerase chain reaction
EMT	Epithelium–mesenchymal transition
GAPDH	Glyceraldehyde 3-phosphate dehydrogenase
mRNA	Messenger RNA
PCA	Principal component analysis
gDNA	Genomic DNA
WNT	Wingless-related integration site
